# Direct Flow Medical vs. Edwards Sapien 3 Prosthesis: A Propensity Matched Comparison on Intermediate Safety and Mortality

**DOI:** 10.3389/fcvm.2021.671719

**Published:** 2021-06-18

**Authors:** Christoph Edlinger, Marwin Bannehr, Bernhard Wernly, Tanja Kücken, Maki Okamoto, Michael Lichtenauer, Valentin Hähnel, David Reiners, Michael Neuss, Christian Butter

**Affiliations:** ^1^Department of Cardiology, Heart Center Brandenburg, Bernau/Berlin, Germany; ^2^Brandenburg Medical School (MHB) “Theodor Fontane”, Neuruppin, Germany; ^3^Clinic of Internal Medicine II, Department of Cardiology, Paracelsus Medical University of Salzburg, Salzburg, Austria; ^4^Faculty of Health Sciences Brandenburg, Brandenburg Medical School Theodor Fontane, Brandenburg, Germany; ^5^Department of Anaesthesiology, Perioperative Medicine and Intensive Care Medicine, Paracelsus Medical University of Salzburg, Salzburg, Austria

**Keywords:** aortic stenosis, TAVI, direct flow medical, Edwards Sapien 3, mortality, MACE, survival

## Abstract

**Aims:** To compare intermediate performance and mortality rates in patients, who underwent transcatheter aortic valve implantation (TAVI) with two different types of prostheses: Edwards Sapien 3 (ES3) and Direct Flow Medical (DFM).

**Methods and Results:** 42 consecutive patients implanted with a DFM prosthesis for severe aortic stenosis were matched 1:1 with an equal number of patients, who received an ES3 during the same period. Primary endpoint was mortality. MACE, as a composite of all-cause death, stroke, and re-do-procedure (valve-in-valve), was defined as secondary endpoint. Moreover, we compared NYHA class, NT-proBNP-levels and the extent of restenosis. Patients were followed for 2 years. DFM patients showed echocardiographic elevated mean pressure gradients compared to ES3 patients before discharge (11.2 mmHg ± 5.3 vs. 3.5 mmHg ± 2.7; *p* < 0.001) and upon 6-months follow-up (20.3 mmHg ± 8.8 vs. 12.3 mmHg ± 4.4; *p* < 0.001). ES3 candidates showed superior NYHA class at follow-up (*p* = 0.001). Kaplan-Meier analysis revealed significantly worse survival in patients receiving a DFM prosthesis compared to ES3 (Breslow *p* = 0.020). MACE occurred more often in DFM patients compared to ES3 (Breslow *p* = 0.006).

**Conclusions:** Patients receiving DFM valve prostheses showed worse survival and higher rates in MACE compared to ES3. Prosthesis performance regarding mean pressure gradients and patients' NYHA class also favored ES3.

## Introduction

Transfemoral aortic valve implantation (TAVI) represents an outstanding success story in interventional cardiology in recent years. Since the first implantation by Cribier et al. (1), there has been an enormous increase in clinical and scientific experience, and the method now represents an indispensable standard therapy (1–4).

Over the years, different types of prostheses have been established as most frequently implanted products worldwide: Namely, the self-expanding Medtronic Corevalve prosthesis (MVC) (Medtronic Inc. Minneapolis, MN, USA) and the balloon-expanding Edwards Sapien 3 prosthesis (ES3) (Edwards Life Sciences Inc. Irvine, CA, USA). In addition, there is a certain number of other models with various potential advantages, such as the Direct Flow medical prosthesis (DFM) (DFM Santa Rosa, CA, USA). This type of prosthesis was intended to offer safety improvements due to its non-metallic construction, but above all due to its ability to inflate and deflate, allowing the system to be repositioned and retrieved during the intervention, if necessary, in order to figure out an optimal position.

Although data in short-term treatment has been very promising, especially concerning lower rates of post-interventional pacemaker implantation, the product did not achieve a sustainable breakthrough and was consequently withdrawn from the market (5, 6). Regarding the direct comparison of the DFM prosthesis only comparative data on short-term treatment exist, whereas little is known about intermediate safety and mortality (7). Nevertheless, a considerable number of patients who have been implanted with this type of prosthesis are still encountered in clinical follow-ups. Since its introduction in 2013 until its withdrawal from the market in late 2016 ~3.000 DFM protheses have been implanted worldwide (32, 33). With an aggregated survival of TAVI patients at 1-, 2-, 3-, and 5-years being 83, 75, 65, and 48%, respectively, numerous patients implanted with DFM valves are considered to be still alive (8).

Now that there is a considerable amount of intermediate data in the field of TAVI, the question of intermediate performance is becoming increasingly important.

It could be shown that patients with paravalvular regurgitation have a significantly worse outcome (9). Kang et al. recently presented the Dicrotic AR Index, a hemodynamic tool for the prediction of paravalvular regurgitation following TAVI (10). How to deal with repeatedly increased gradients, on the other hand, seems particularly challenging, since an increase in mean pressure gradients (PGmean) may also be related to a post-interventional improvement of left ventricular function. Thus, in contrast to conventional aortic valve replacement, there are currently no reference values from which a regular TAVI function can be conclusively assessed. Protheses degeneration is a known problem that occurs over the years. However, little has been reported with regard to DFM valves in particular.

In our current study we investigated the intermediate course of patients, who had been implanted with a DFM and those who received an ES3, based on a 1:1 propensity matched score. We compared performance, safety and overall all-cause mortality.

## Methods

### Study Design

**Figure 1:**
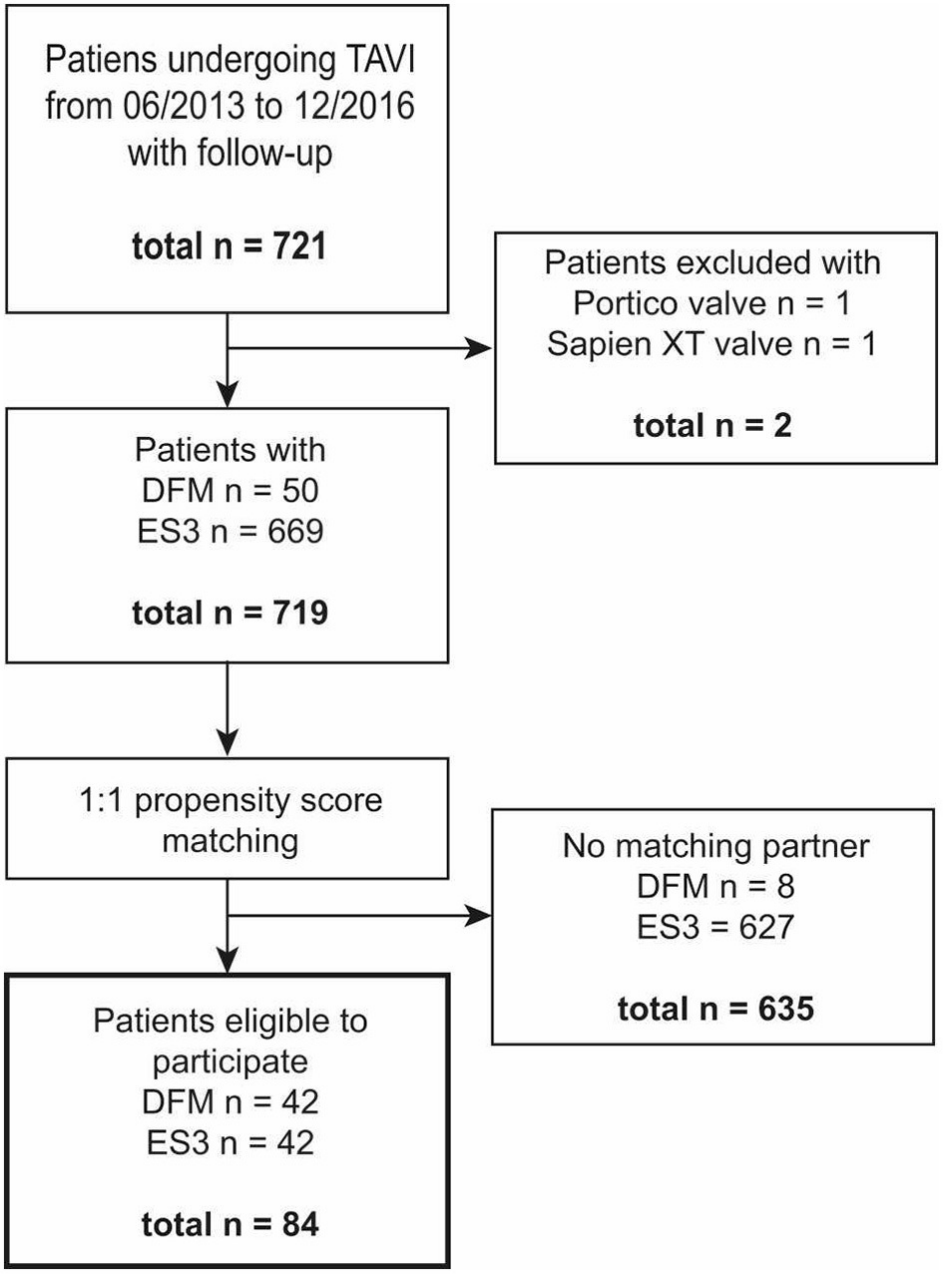
Patient flow through the study. Figure shows patients included and excluded in the study with a number of *n* individuals at each step. TAVI, transcatheter aortic valve replacement; DFM, direct flow medical prosthesis; ES 3, Edwards Sapien 3 prothesis.

In this retrospective observational comparative-cohort study we analyzed the clinical course of patients who were treated at our center from 06/2013 to 12/2016. A total number of 721 individuals could be identified within this period. Of these, each received either a DFM or an ES3 prosthesis. Patients were followed for 2-year survival. Fifty patients who received a DFM were matched 1:1 with those who received an ES3. Within the DFM group, eight patients had to be excluded because no appropriate matching partner could be identified. Thus, a total of 84 patients were included in our analyses as the defined study population (Figure 1).

The case of each individual patient was discussed in advance by our interdisciplinary heart team, consisting of cardiologists and heart surgeons, where the consensus for a TAVI procedure had been set.

All patients underwent transthoracic echocardiography, in order to confirm the diagnosis. Moreover, a multi-slice computed tomography was performed for assessment of dimensions and morphology of the aortic valve and aorta. The estimation of surgical risk was performed by using the EuroScore (11). The definition of severe aortic stenosis was made according to the current guideline of the European Society of Cardiology (PGmean >40 mmHg or peak jet velocity >4.0 m/s or aortic valve area </=0.8 cm^2^ or aortic valve area index <0.5 cm^2^/m^2^) (12).

The decision on the type of prosthesis to be implanted was made for each individual case by the performing interventionalist. Clinical prerequisites (NYHA class, LVEF, etc.) were identical. The rationale for valve selection was based on anatomical features in pre-procedural multi slice computed tomography imaging. Patients who showed complicating anatomical conditions (asymmetry, spur of calcification protruding into the outflow tract or bicuspid valve) were not suitable for a DFM prothesis. For the purpose of quantification of valvular calcification, the aortic root was separated into three regions along its long axis: annulus, leaflets, and left ventricular outflow tract. Three mensio Valves software (PIE Medical Imaging, Maastricht, Netherlands) was used to measure calcification with a 550 HU threshold.

Prior written consent was obtained from all patients both for the TAVI procedure and for the use of data.

The study was conducted in accordance with the guidelines of the Declaration of Helsinki.

### Data Collection

Demographics and clinical data including relevant comorbidities and laboratory results were collected using patient medical records. Follow-up data for survival rates were collected from hospital database and primary care physicians via telephone interview. Patients were followed for all-cause mortality.

Echocardiographic examinations were performed with commercially available ultrasound systems (GE-Vingmed, Vivid 7 and E9, Horten Norway) by trained physicians.

### Statistical Analysis

Data was analyzed using descriptive statistics, with categorical variables presented as absolute values and frequencies (%) and continuous variables presented as means with standard deviation or median and interquartile range (IQR) when appropriate.

A propensity score was calculated using logistic regression from the covariates Euroscore, body mass index (BMI), creatinine, left ventricular ejection fraction (LVEF), PGmean, and N-terminal pro brain natriuretic peptide (NT-proBNP). A matched cohort was obtained using the “nearest neighbor” method. The maximum allowed distance was a Δ in propensity score of 0.001. Since the variable age is already included in the EuroScore as a continuous value it has not been taken into account for matching. Comparisons between groups were carried out using Student's *t*-test for continuous variables and a Mann-Whitney-*U*-test for categorical variables. Survival analysis data is presented as Kaplan-Meier curves. A two-tailed *p*-value of <0.05 was considered statistically significant. All statistical tests were performed using IBM SPSS Statistics software version 24.0 (IBM Corporation, Armonk, NY, USA).

### Prosthesis Types

The DFM prosthesis was a percutaneous valve with a non-metallic design. The valve apparatus itself is based on bovine pericardium (13). The basic structure of the prosthesis is made of dacron/polyester, whereby two rings have been designed of which the upper ring is positioned in the aorta and the lower ring on the side of the ventricle. Both rings can be individually controlled and unfolded.

A total of four sizes were available (23/25/27/29 mm).

Immediately after the balloon valvuloplasty, the valve was positioned using the guiding catheter. The rings were first filled with a saline solution to a pressure of 12 ATM and could be readjusted, if necessary, until the desired position was reached. Once the ideal position had been found, the saline solution was exchanged for a polymer while maintaining the pressure, which led to the hardening of the prosthesis in position (14).

The ES3 has already been well-described elsewhere (15–17). In brief, it is a balloon expanding valve of the second generation. The frame is made of cobalt, whereas the valve apparatus is made of bovine pericardium. This type of prosthesis is available in four sizes (20/23/26/29 mm).

## Results

A total of 84 patients were included in this study. Patients were at advanced age and a majority obese with numerous comorbidities as typical for a TAVI cohort. Table 1 provides a detailed overview of patient characteristics before and after propensity score matching. While patients who received a DFM showed lower EuroScore, impaired LVEF, better renal function, and a higher prevalence of cardiac devices, there were no significant differences between the two groups after matching, with regard to baseline characteristics.

**Table 1:** Patients' baseline characteristics.

	**Matched cohort**					**Unmatched cohort**				
	**Overall *n* = 84**	**DirectFlow *n* = 42**	**Edwards Sapien 3 *n* = 42**	***P*-value**	**SDM**	**Overall *n* = 719**	**DirectFlow *n* = 50**	**Edwards Sapien 3 *n* = 669**	***P*-value**	**SDM**
BMI (kg/m^2^)	*27.9 ± 4.6*	*27.7 ± 4.9*	*28.2 ± 4.3*	*0.637*	*−0.11*	*27.7 ± 5.4*	*27.0 ± 5.1*	*28.3 ± 5.7*	*0.104*	*0.04*
Log. EuroScore I	*15.5 ± 11.2*	*15.7 ± 6.8*	*15.3 ± 14.4*	*0.869*	*0.04*	*19.2 ± 6.6*	*16.2 ± 7.1*	*22.1 ± 6.0*	0.018^*^	*−0.56*
PGmean prior TAVI (mmHg)	*41.4 ± 17.4*	*42.0 ± 16.1*	*40.1 ± 18.8*	*0.770*	*0.11*	*40.9 ± 16.8*	*40.8 ± 16.4*	*40.9 ± 16.9*	*0.960*	*0.03*
LV-EF (%)	55.4 ± 9.7	55.2 ± 10.5	55.7 ± 9.1	0.807	−0.05	49.2 ± 10.4	53.3 ± 11.8	45.1 ± 8.9	0.028^*^	0.59
Creatinine (μmol/l)	90.8 ± 21.7	*92.6 ± 21.7*	*89.1 ± 21.8*	*0.457*	*0.16*	*109.2 ± 72.9*	*93.3 ± 21.9*	*110.4 ± 75.3*	<0.001^*^	*−0.33*
NT-proBNP prior TAVI (pg/ml)	*3,534 ± 5,056*	*3,750 ± 5,660*	*3,317 ± 4,430*	*0.697*	*0.09*	*4,766 ± 6,802*	*3,788 ± 5,343*	*4,841 ± 6,898*	*0.292*	*−0.19*
Sex male (%)	36.9	45.2	28.6	0.113		51.7	50.0	52.1	0.392	
Age (years)	81.2 ± 5.6	81.7 ± 5.2	80.7 ± 5.9	0.413		80.2 ± 6.2	81.7 ± 5.0	80.1 ± 6.2	0.065	
PCI prior TAVI (%)	36.9	28.6	45.2	0.113		41.3	30.0	42.7	0.052	
CABG prior TAVI (%)	6.0	7.1	4.8	0.645		11.0	10.0	11.2	0.511	
ICD or pacemaker prior TAVI (%)	11.2	7.9	14.3	0.322		13.8	6.0	14.7	0.034^*^	
Type II diabetes (%)	36.9	35.7	38.1	0.821		41.4	36.0	42.5	0.228	

*SDM, standardized mean difference; PCI, percutaneous coronary intervention; CABG, coronary artery bypass craft; GFR, glomerular filtration rate; LV-EF, left ventricular ejection fraction*.

*Matching variables are shown in italics and are reported with SDM.*

* *p-values*.

Calcium volume on computed tomography differed between groups with respect to valvular leaflets (415.9 ± 243.0 vs. 247.3 ± 182.7 mm^3^; *p* = 0.001) with a higher calcium load in the DFM group. LVOT and annular calcium did not differ significantly (Table 2).

**Table 2:** Volume of calcification (matched cohort).

	**Overall**	**DirectFlow**	**Edwards Sapien 3**	***P*-value**
LVOT Calcium (mm^3^)	8.1, 17.7	10.6, 21.3	5.4, 12.7	0.210
Annulus Calcium (mm^3^)	41.8, 51.3	47.9, 60.9	35.4, 38.6	0.309
Leaflet Calcium (mm^3^)	333.9, 230.5	415.9, 243.0	247.3, 182.7	0.001^*^

* *p-values*.

TAVI performance differed significantly in regard to both prosthesis types: Patients who received DFM showed increased PGmean in transthoracic echocardiography compared to ES3, both before discharge from hospital (11.2 ± 5.3 vs. 3.5 ± 2.7; *p* ≤ 0.001) and at 6-month follow-up (20.3 ± 8.8 vs. 12.3 ± 4.4; *p* < 0.001) (Table 3).

**Table 3:** TAVI performance-Comparison DirectFlow vs. Edwards Sapien 3 (matched cohort).

	**Overall**	**DirectFlow**	**Edwards Sapien 3**	***P*-value**
Valve size	N/A	23 mm *n* = 1 25 mm *n* = 21 27 mm *n* = 19 29 mm *n* = 1	23 mm *n* = 17 26 mm *n* = 20 29 mm *n* = 5	N/A
PGmean prior TAVI (mmHg)	41.4 ± 17.4	42.0 ± 16.1	40.1 ± 18.8	0.770
PGmean post TAVI (mmHg)	6.8 ± 5.5	11.2 ± 5.3	3.5 ± 2.7	<0.001^*^
PGmean at 6-months FU TAVI (mmHg)	16.2 ± 7.9	20.3 ± 8.8	12.3 ± 4.4	<0.001^*^
NYHA at admission (%)				0.097
II	13.0	10.5	15.4	
III	77.9	73.7	82.1	
IV	9.1	15.8	2.6	
NYHA at 6-months FU (%)				<0.001^*^
II	41.8	20.5	52.5	
III	58.2	79.5	37.5	
IV	0	0	0	
NT-proBNP prior TAVI (pg/ml)	1.819 (IQR 3.498)	1.840 (IQR 2.631)	1.669 (IQR 3.689)	0.847
NT-proBNP at discharge (pg/ml)	1.576 (IQR 2.740)	2.230 (IQR 2.986)	1.169 (IQR 2.296)	0.226
NT-proBNP at 6-months FU (pg/ml)	633 (IQR 1.439)	624 (IQR 2.909)	681 (IQR 991)	0.414
Post-procedural AR/PVL (%)	44.0	42.9	45.3	0.933
Minimal	21.4	16.7	26.2	
Mild	17.9	21.4	14.3	
Moderate	4.8	4.8	4.8	
PPI post TAVI (%)	13.1	7.1	19.0	0.116
Post-procedural LBBB TAVI (%)	15.5	14.3	16.7	0.500
Hospital stay (days)	10.9 ± 6.2	10.6 ± 6.1	11.2 ± 6.3	0.676
Valve in valve re-do procedure (*n*)	4	3	1	0.308
MACE (*n*)	12	10	2	0.013^*^
All-cause death (*n*)	10	8	2	0.044^*^
Stroke (*n*)	2	2	0	0.247

*PG, pressure gradient; TAVI, transcatheter aortic valve implantation; NYHA, New York Health Association functional class; FU, follow-up; AR, aortic regurgitation; PVL, paravalvular leakage; PPI, permanent pacemaker implantation; LBBB. left bundle branch block; MACE, major adverse cardiac event*.

* *p-values*.

Patients improved in NYHA functional class in both groups at 6-months compared to hospital admission. Patients receiving an ES3 were superior compared to DFM (Table 3).

Overall median NT-proBNP levels declined within 6 months following TAVI (1.819 pg/ml [IQR 3.498] vs. 633 pg/ml [IQR 1.439]). There was no statistically significant difference between groups, while there was a trend toward lower levels in those with ES3 (Table 3).

None of the calcification variables assessed were predictive of having a post-procedural prothesis PGmean >20 mmHg. Leaflet calcium volume correlated only weakly with post-procedural PGmean (*r* = 0.27, *p* = 0.029).

Residual aortic regurgitation and/or paravalvular leakage did not differ between groups (Table 3).

Permanent pacemaker implantation was necessary in 7.1% individuals with a DFM prothesis compared to 19.0% with an ES3 (*p* = 0.116). New complete left bundle branch block occurred in 14.3% (DFM) vs. 16.7% (ES3) of patients (*p* = 0.500).

Three patients underwent valve in valve re-do procedure in the DFM group compared one in the ES3 group. Duration of hospital stay did not differ between groups (Table 3). Patients presented again with symptomatic heart failure and markedly elevated pressure gradients. None of the four cases were reintervened because of paravalvular leakage or relevant aortic regurgitation.

Thrombocyte aggregation inhibitors post TAVI did not differ between groups. While a significant number of patients received direct oral anticoagulants in the ES3 group there were more patients discharged on low molecular heparin in the DFM group (Table 4).

**Table 4:** Anticoagulants and thrombocyte aggregation inhibitors post TAVI (matched cohort).

	**Overall**	**DirectFlow**	**Edwards Sapien 3**	***P*-value**
ASS (%)	56.8	60.0	53.7	0.567
Clopidogrel (%)	88.9	95.0	82.9	0.086
Ticagrelor (%)	1.2	0	2.4	0.323
Prasugrel (%)	0	0	0	N/A
DOAK (%)	6.2	0	12.2	0.024^*^
Apixaban (%)	1.2	0	2.4	
Rivaroxaban (%)	3.7	0	7.3	
Dabigatran (%)	0	0	0	
Edoxaban (%)	1.2	0	2.4	
Vitamin K antagonist	37.0	37.5	36.6	0.933
Low molecular heparin	30.9	35.0	26.8	0.429
Fondaparinux	1.2	0	2.4	0.323

*Standard thrombocyte aggregation inhibitor regimen was ASS plus Clopidogrel for 3–6 months post-TAVI unless there was an indication for anticoagulation (in patients with atrial fibrillation, thrombosis, or pulmonary embolism), another thrombocyte aggregation inhibitor regimen required according to guidelines (prior myocardial infarction, previous coronary intervention in Clopidogrel non-responders), or an allergic reaction to one of the substances. Patients with indication for anticoagulation received vitamin K antagonist plus Clopidogrel for 3–6 months as first line therapy. DOAK, direct oral anticoagulant*.

* *p-values*.

### Survival Analysis

Out of all 84 patients 10 died within the 2-year follow-up period (8 DFM, 2 ES3).

**Figure 2:**
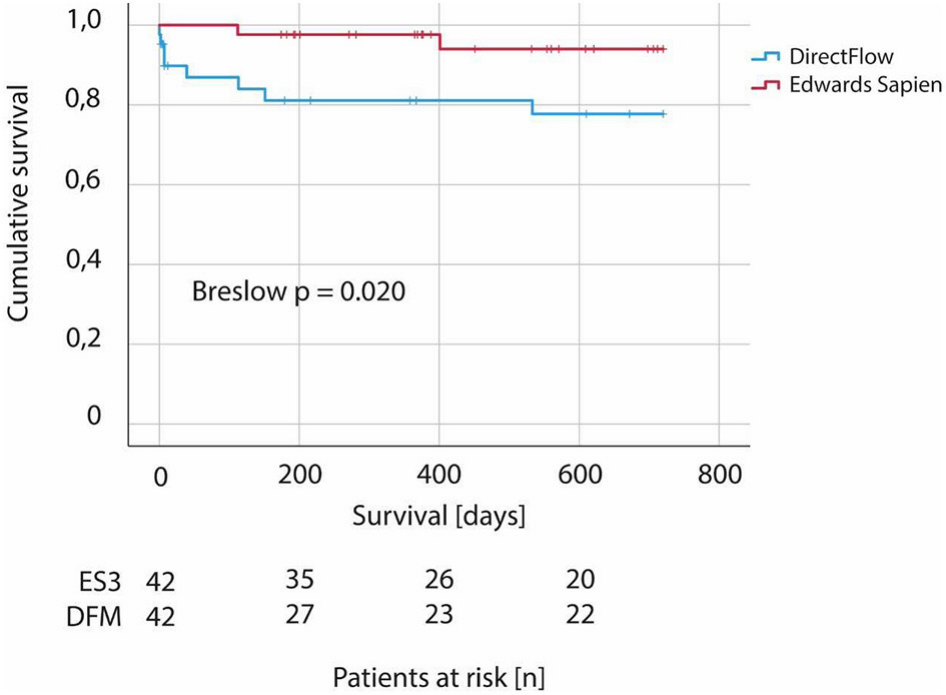
Two-year survival in patients undergoing TAVI—comparison between DirectFlow and Edwards Sapien 3. Kaplan-Meier analysis according to type of valve (DirectFlow vs. Edwars Sapien 3). *N* = 84.

As shown in Figure 2 Kaplan-Meier survival analysis showed significantly worse survival in patients receiving a DFM prosthesis compared to ES3 (Breslow *p* = 0.020).

**Figure 3:**
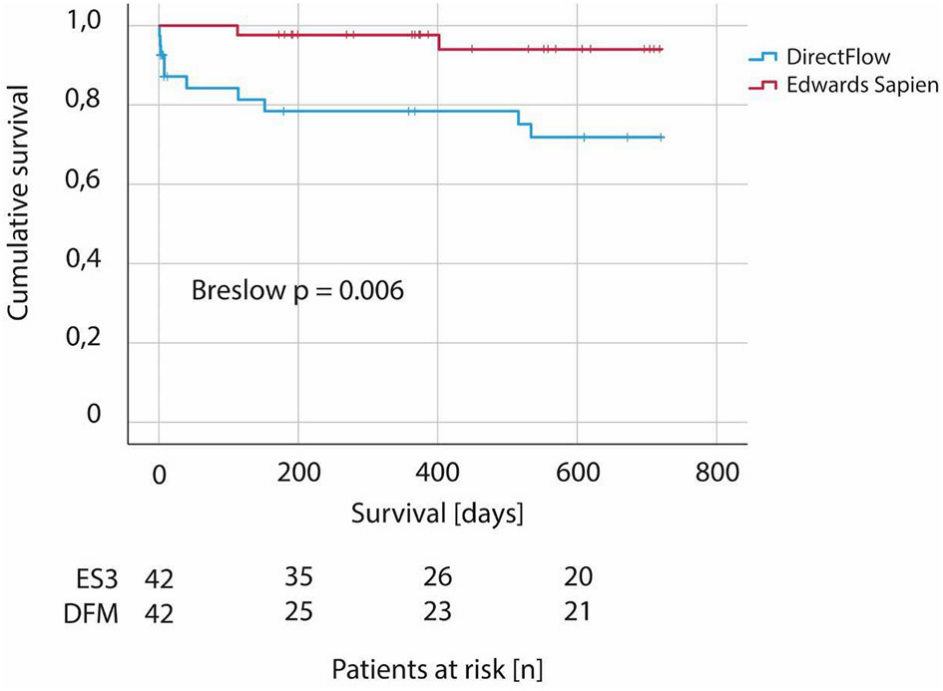
Two-year MACE in patients undergoing TAVI—comparison between DirectFlow and Edwards Sapien 3. MACE defined as composite of all-cause death, stroke, and re-do-procedure (valve-in-valve) within 2 years from implantation of first valve (DirectFlow vs. Edwars Sapien 3). *N* = 84.

MACE, as a composite of all-cause death, stroke, and re-do-procedure (valve-in-valve), occurred more often if a DFM was the prosthesis of choice compared to ES3 (10 DFM, 2 ES3; Breslow *p* = 0.006) (Figure 3).

## Discussion

To the best of our knowledge, this is the first study to compare the intermediate performance of DFM and ES3 valve prostheses by using propensity score matching. Our findings can be summarized as follows:

1. Patients with DFM have a higher risk of death within 2 years compared to ES3.
2. Patients with DFM have a significantly higher risk of suffering a MACE [composed of all-cause death, stroke, and re-do-procedure (valve-in-valve)].
3. At 6-months follow-up patients with DFM showed a significantly higher PGmean.
4. The NYHA class was significantly worse in patients with DFM at 6-months follow-up, while differences in NT-proBNP levels were not of statistical significance.

While previous studies could document an equivalent performance between the prosthesis types within the early phase of therapy, we saw a significant increase in all-cause mortality in patients who primarily received a DFM within our propensity score matched study population (5, 6, 14).

At 6-months follow-up already, we could observe significantly increased pressure gradients in DFM patients. Especially since we also observed higher NYHA classes at this time, we assume that there is a significantly worse hemodynamic performance, which apparently already occurs after only a few months. This significant hemodynamic disadvantage may have a substantial impact on the patients' clinical benefit and ultimately result in a significantly higher mortality in DFM compared to ES patients.

It may sound tempting to have a prosthesis available, that is both non-metallically designed and that can be repositioned, the product obviously has some weak points. If an increased gradient has been detected, one is initially inclined to think of a thrombogenic etiology first. According to current data however, manifest thromboses of the valves leading to clinical symptoms are a rare event after TAVI (18). In contrast, partial thromboses are encountered significantly more frequently, which usually do not have a clinical impact (19). Since we did not have any relevant differences in coagulation management within the two groups, we consider a thrombotic etiology unlikely.

On the one hand, we assume that the material used might itself be more prone to faster degeneration. Comparable data on primary degeneration of that part of the valve made from animal pericardium exist from conventional aortic valve replacement (20–23). When these processes begin and when clinical relevance is reached remains to be determined in regard to TAVI, at least in the case of prostheses of the latest generation (24–26).

On the other hand, the DFM prosthesis cannot be inserted into the left ventricular outflow tract with the same pressure as the ES3 or other metal-based products, so the effective opening area might be smaller right from the start. We assume that hemodynamically, an effect comparable to that known from conventional surgical aortic valve replacement in case of a prosthesis mismatch could occur.

A basic difference can already be found in the selection of the size of the prosthesis. In principle, the balloon-expandable ES3 valve is selected according to the annular area, not the circumference (i.e. perimeter); whereas the DFM is selected according to the perimeter—not the area. If one calculates “roughly,” the S3 23 mm is implanted at a perimeter of 75–83 mm, the S3 26 mm and DFM Flow 27 mm for a perimeter of 79–85 mm. In the end, this difference is reflected in the effective opening area of the DFM. According to current literature mean opening area is 1.6 cm^2^ ± 0.4 and thus the same size as smallest version of ES3 (23 mm) (27, 28).

An interesting side finding of our work is the fact that the three patients in the DFM group with a reasonable clinical intermediate outcome had undergone a “valve in valve” procedure. Already in 2016, our group was able to publish the world's first case of a “valve in valve” implantation after DFM implantation (29). Furthermore, Yap et al. and Karaduman et al. independently reported on the technical challenges of a valve in valve procedure when the first prosthesis was a DFM recently (30, 31).

How many of the patients implanted with a DFM prosthesis are actually still alive can only be estimated to a certain extent. An official announcement by the manufacturer states that a total of 2.700 prostheses had been implanted by May 2016 (33). According to the review by Chakos et al. aggregated survival at 1-, 2-, 3-, and 5-years after TAVI were 83, 75, 65, and 48%, respectively, for different manufacturers (8).

Thus, it can be concluded that a considerable number are still alive. It seems important to us to follow up these patients carefully and to evaluate whether a “valve-in-valve” procedure can be considered in the case of symptomatic restenosis.

In our cohort two patients who initially received a DFM 25 mm could be fitted with an ES XT 20 mm and Medtronic Evolut R 23 mm, one patient who initially had a DFM 27 mm implanted could also be treated with a Medtronic Evolut R 23 mm. In all cases, the intervention proved to be safe and without complications. For this reason, we assume that patients with DFM can undergo a valve in valve procedure in an experienced center with an acceptable risk, so that at least a therapeutic perspective can be offered to patients with a degenerated DFM prosthesis.

## Limitations

The main limitations of our study are its retrospective, non-randomized design, and the single center setting. There was only a limited number of patients who died within the study period. Nevertheless, due to the significant differences in both, mortality and restenosis rates, we are convinced that the conclusions drawn are valid despite the relatively low number of cases. Since the TAVI population is generally an old, correspondingly pre-diseased patient population, natural patient death must be included in the consideration. The first DFM patients within our cohort were implanted as of June 2013. The market launch of the ES3 did not take place until January 2014, so that there is no exact time overlap between the two groups. Propensity score matching did not include prothesis size since direct comparison did not seem appropriate. However, as shown in Table 3, there were rather more patients in the ES3 group with smaller protheses, thus size (diameter) itself should not account for the differences we observed.

## Conclusion

Patients receiving a DFM valve prosthesis showed worse survival and higher rates in MACE compared to ES3. Valve performance regarding mean pressure gradients and patients' NYHA class also favored ES3. Valve in valve re-do procedures showed to be safe in DFM patients as a bail out option for a degenerate prosthesis.

## Data Availability Statement

The original contributions presented in the study are included in the article/supplementary material, further inquiries can be directed to the corresponding author/s.

## Ethics Statement

The study was performed in accordance with the Declaration of Helsinki. The ethics committee advised that no formal vote and no written informed consent beyond the agreement at hospital admission was necessary because data collection and assessment was part of hospital-wide measure of quality management.

## Author Contributions

CE coordinated the study, acquisited and analyzed data, wrote the manuscript, and contributed in the final submission. MB analyzed data, prepared figures, and contributed in manuscript preparation. BW analyzed data and contributed in final submission. TK, MO, VH, and DR contributed in data acquisition. ML and MN revised the article critically for the content. CB planned the study and provided final approval of the article. All authors contributed to the article and approved the submitted version.

## Conflict of Interest

The authors declare that the research was conducted in the absence of any commercial or financial relationships that could be construed as a potential conflict of interest.
